# Cost-effectiveness of dapagliflozin versus DPP-4 inhibitors as an add-on to Metformin in the Treatment of Type 2 Diabetes Mellitus from a UK Healthcare System Perspective

**DOI:** 10.1186/s12913-015-1139-y

**Published:** 2015-11-05

**Authors:** M. Charokopou, P. McEwan, S. Lister, L. Callan, K. Bergenheim, K. Tolley, R. Postema, R. Townsend, M. Roudaut

**Affiliations:** Pharmerit International, Rotterdam, Netherlands; Centre for Health Economics, Swansea University, Swansea, UK; HEOR, Monmouth, UK; Bristol-Myers Squibb Pharmaceuticals Ltd, Uxbridge, UK; AstraZeneca UK Ltd, Luton, UK; AstraZeneca, Molndal, 431 83 Sweden; Tolley Health Economics Ltd., Buxton, UK; Bristol-Myers Squibb, Rueil-Malmaison, France; AstraZeneca, Brussels, Belgium

**Keywords:** SGLT 2, DPP-4i, Type 2 diabetes mellitus, Cost-effectiveness analysis

## Abstract

**Background:**

Type 2 diabetes mellitus (T2DM) is a chronic, progressive condition where the primary treatment goal is to maintain control of glycated haemoglobin (HbA1c). In order for healthcare decision makers to ensure patients receive the highest standard of care within the available budget, the clinical benefits of each treatment option must be balanced against the economic consequences.

The aim of this study was to assess the cost-effectiveness of dapagliflozin, the first-in-class sodium-glucose co-transporter 2 (SGLT2) inhibitor, compared with a dipeptidyl peptidase-4 inhibitor (DPP-4i), when added to metformin for the treatment of patients with T2DM inadequately controlled on metformin alone.

**Methods:**

The previously published and validated Cardiff diabetes model was used as the basis for this economic evaluation, with treatment effect parameters sourced from a systematic review and network meta-analysis. Costs, derived from a UK healthcare system perspective, and quality-adjusted life years (QALYs), were used to present the final outcome as an incremental cost-effectiveness ratio (ICER) over a lifetime horizon. Univariate and probabilistic sensitivity analyses (PSA) were carried out to assess uncertainty in the model results.

**Results:**

Compared with DPP-4i, dapagliflozin was associated with a mean incremental benefit of 0.032 QALYs (95 % confidence interval [CI]: −0.022, 0.140) and with an incremental cost of £216 (95 % CI: £-258, £795). This resulted in an ICER point estimate of £6,761 per QALY gained. Sensitivity analysis determined incremental costs to be insensitive to variation in most parameters, with only the treatment effect on weight having a notable impact on the incremental QALYs; however, there were no scenarios which raised the ICER above £15,000 per QALY. The PSA estimated that dapagliflozin had an 85 % probability of being cost-effective at a willingness-to-pay threshold of £20,000 per QALY gained.

**Conclusions:**

Dapagliflozin in combination with metformin was shown to be a cost-effective treatment option from a UK healthcare system perspective for patients with T2DM who are inadequately controlled on metformin alone.

## Background

Type 2 diabetes mellitus (T2DM) is a chronic condition characterised by elevated blood glucose levels as a result of resistance to the action of insulin. T2DM can lead to numerous micro- and macro-vascular complications and may cause substantial disability. It is increasingly prevalent, with the T2DM population in the UK expected to rise to 3 million by 2017 [1], and it is currently estimated to account for 7–12 % of the total UK National Health Service (NHS) expenditure [2, 3]. Although drug costs are increasing [1], the greatest component of the economic burden of T2DM is the treatment of diabetic complications [2], which can be reduced with effective management of the disease.

The primary treatment goal of T2DM management is to reduce glycated haemoglobin (HbA1c) levels to below 6.5 % for first line treatment or below 7.5 % for second line treatment. This is recommended in the UK by the National Institute for Health and Care Excellence (NICE) in order to effectively reduce diabetes-related complications [3]. The principles of the NICE guidelines are in line with those outlined in the American Diabetes Association (ADA) and the European Association for the Study of Diabetes (EASD) combined position statement, which support a target HbA1c goal for adults with T2DM of around 7 %, depending on individual patient characteristics [4]. However, T2DM represents a major clinical priority, as between 30–40 % of all patients receiving treatment fail to reach the blood glucose targets recommended by NICE and over three-quarters are overweight or obese [4, 5].

Metformin is commonly used as a first-line treatment in diabetes; however, due to the progressive nature of T2DM, many patients at some point will require additional therapy to maintain glycaemic control. The selection of additional treatment options is often complex due to the number of factors that must be considered. Unintended sequelae such as hypoglycaemia, weight changes and side effects are important considerations as they can have a significant impact on patients’ adherence and quality of life [4].

Dapagliflozin was the first in a new class of selective sodium-glucose co-transporter 2 (SGLT2) inhibitors licensed in Europe. Both dapagliflozin and dipeptidyl peptidase-4 inhibitors (DPP-4i) have been recommended by NICE in the UK as second-line therapies (dual therapy, add-on to metformin) in patients with T2DM, when diet and exercise plus metformin fail to achieve glycaemic targets. In order for healthcare decision makers to ensure patients receive the highest standard of care within the available budget, the clinical benefits of each treatment option must be balanced against the economic consequences. This study aimed to assess the long-term cost-effectiveness of dapagliflozin versus DPP-4i, as dual oral therapies in combination with metformin, in patients who were inadequately controlled on metformin alone, from the perspective of the UK NHS. The objective was to present the model here as it was reviewed and accepted by NICE.

In addition to glycaemic control, key factors that may differ across therapies and therefore drive treatment decisions in clinical practice, such as weight and hypoglycaemic risk, were also considered in the analysis. Results of a previously published network meta-analysis (NMA), comparing the major clinical outcomes for dapagliflozin with DPP-4i as an add-on to metformin [6], acted as a key source of clinical inputs for this economic analysis. This reported a non-significant reduction in HbA1c (−0.08 % [95 % CI: −0.25, 0.10]) and a significant reduction in weight (−2.85 kg [95 % CI: −3.39, −2.30]) for dapagliflozin compared with DPP-4i [6]. Assessments of the cost-effectiveness of dapagliflozin versus other antidiabetec agents used as add-ons to metformin [7], for indications other than as an add-on to metformin and in settings other than the UK [8] have been presented elsewhere.

## Methods

The economic evaluation analysed the cost-effectiveness of dapagliflozin as an add-on to metformin (DAPA + MET) versus DPP-4i as an add-on to metformin (DPP-4i + MET) in adults aged 18 years and older with T2DM who were inadequately controlled on metformin alone. The main assessment metric was the incremental cost-effectiveness ratio for dapagliflozin compared with DPP-4i therapy, with effectiveness measured in quality-adjusted life years (QALYs). QALYs represent a composite measure of estimated post-treatment life years adjusted for the quality of life (or utility) of those life years. The economic evaluation was conducted from the perspective of the UK NHS, and a discount rate of 3.5 % was applied to both costs and health effects as recommended in the NICE Methods Guide [9].

### Model structure

The published Cardiff stochastic simulation diabetes model was used as the basis for this economic evaluation as this has previously been validated to accurately model important clinical outcomes for diabetic patients [10–12]. The model utilises risk equations from the UK Prospective Diabetes Study (UKPDS) 68 to estimate long-term micro- and macro-vascular complications, as well as diabetes-related mortality and non-diabetes-related mortality [13]. In total, seven micro- and macro-vascular complications were included in the model (ischaemic heart disease, myocardial infarction, congestive heart failure, stroke, amputation, blindness, and end-stage renal disease), along with cardiovascular (CV) death, non-CV death, drug-related hypoglycaemic events and additional adverse events associated with an SGLT2 inhibitor. The cumulative incidence of all complications depended on patients’ baseline characteristics and time- and treatment-dependent evolution of modifiable risk factors, including HbA1c, body mass index (BMI), the ratio of total- and high-density lipoprotein (HDL) cholesterol and systolic blood pressure (SBP). This allowed the model to measure the extent to which age and gender affected the incidence of diabetes-related complications and to take into account factors such as the increased risk of stroke amongst smokers and the lower incidence of MI associated with the UK Afro-Caribbean population with T2DM [13].

In the base case analysis, 100 cohorts of 30,000 individual patients were modelled and this was tested to ensure stability in the simulation results had been reached. Patients were simulated through 6-monthly time intervals over a total period of 40 years, indicative of a lifetime horizon for an average T2DM patient. Six-monthly rather than annual cycles were chosen to allow more detailed transitions to be modelled and to reflect the common follow-up time in clinical practice. At the end of each 6-month cycle, the UKPDS risk equations determined the occurrence of the fatal and non-fatal complications. Annual UKPDS risk equations were adjusted to reflect 6-monthly risks by converting to a rate and then converting this to a 6-monthly time frame. All-cause mortality events were estimated using gender-specific life tables for the UK [14]. Once a fatal event occured in the model, life years and QALYs were updated and the simulation ended for the patient.

In the model, each treatment would result in a one-year reduction in HbA1c; this timeframe was reflective of the length of data from the NMA data source comparing dapagliflozin with oral antidiabetic therapies [6]. After this point, a continued rise was assumed due to disease progression, which was derived from a regression analysis of the UKPDS dataset [13]. Similar assumptions were used for SBP and cholesterol.

In clinical studies, DAPA + MET has resulted in a statistically significant reduction in body weight compared with a sulphonylurea + MET and with placebo [15, 16]. Significant differences between DAPA + MET and DPP-4i + MET for this outcome were also demonstrated in the NMA [6]. Hence, the effect of patient weight in terms of risk of CV complications and the impact on patient health-related quality of life (HRQoL) was incorporated into the economic model. Initially, progression of weight was established from the impact of each treatment on weight over a 12-month period. In the dapagliflozin arm, patients' weight was assumed to be maintained in year 2 based on 2-year clinical extension data [17]. The same assumption of stable weight in year 2 was also made for the comparator arm. At the time this analysis was performed for NICE, no further long-term data on patient weight were available and therefore the assumption was made that the initial weight loss would be fully regained in a linear manner to a level that corresponded to the patients’ baseline weight. However, a recent study has shown that the weight lowering effect of dapagliflozin is maintained for four years [18], suggesting that the assumptions used in the model were conservative.

### Patient population

The model population was a cohort representative of UK T2DM patients and was designed to best illustrate where dapagliflozin would be used as part of a UK treatment strategy. In line with clinical trials, patients considered in this model had failed to achieve adequate control on prior metformin monotherapy and therefore required modification to their treatment regimen. Baseline characteristics for intervention and comparator arms (Table 1) were sourced from a systematic review and class-level NMA of relevant phase 3 randomised controlled trials (RCTs) [6].

**Table 1:** Summary of model inputs: baseline characteristics and treatment effects

Baseline characteristic		Baseline value		
Age (years)		57.51		
Proportion female (%)		47.00		
Duration of diabetes (years)		6.01		
Height (m)		1.69		
Proportion Afro-Caribbean (%)		6.20^b^		
Proportion smokers		36.90		
HbA1c^a^ (%)		8.05		
Weight (kg)		87.84		
SBP (mmHg)		133.30		
TC (mg/dL)		199.57		
HDL-C (mg/dL)		44.09		
Treatment effect	DAPA + MET	DPP-4i + MET	Insulin + MET^g^	Intensified insulin^c^
∆HbA1c^c^ (%)	−0.69	−0.61	−1.10	−1.11
∆Weight^c^ (kg)	−3.36	−0.61	+1.08	+1.90^h^
∆SBP^c^ (mmHG)	0^d^	0^d^	0^d^	0^d^
∆TC^c^ (mg/dL)	0^d^	0^d^	0^d^	0^d^
∆HDL-C^c^ (mg/dL)	0^d^	0^d^	0^d^	0^d^
Probability of Discontinuation^e^	0.081	0.043	0^d^	0^d^
Probability. of hypoglycaemic events (symptomatic)^f^	0.031	0.046	0.011	0.616
Probability of hypoglycaemia (severe)^f^	0.0004	0.001	0.037	0.022
Probability of urinary tract infection^f^	0.074	0.054	0^d^	0^d^
Probability of genital infection^f^	0.123	0^d^	0^d^	0^d^
Event	Utility decrement		Source	
Diabetes-related complications				
Ischaemic heart disease	0.090		Clarke, 2003 [23]	
Myocardial infarction	0.550		Clarke, 2003 [23]	
Congestive heart failure	0.108		Clarke, 2003 [23]	
Stroke	0.164		Clarke, 2003 [23]	
Amputation	0.280		Clarke, 2003 [23]	
Blindness	0.074		Clarke, 2003 [23]	
End-stage renal disease	0.263		Currie, 2005 [24]	
Hypoglycaemia				
Symptomatic	0.042		Currie, 2006 [39]	
Nocturnal	0.008		Currie, 2006 [39]	
Severe	0.047		Currie, 2006 [39]	
Adverse events				
Urinary tract infection (UTI)	0.00283		Barry, 1997 [25]	
Genital infection	0.00283		Assumed to be the same as UTI	
BMI changes				
Per unit increase	0.0472		Lane, 2012 [26]	
Per unit decrease	+0.0171		Lane, 2012 [26]	
Drug acquisition cost	Price per tablet^i^	Dose per tablet/pen	Daily dose	Annual cost (£)
Dapagliflozin	£1.31	10 mg	10 mg	£476.92
DPP-4i (sitagliptin^j^)	£1.19	100 mg	100 mg	£433.57
Metformin	£0.02	500 mg	2000 mg	£23.46
Insulin^k^ (Insuman® Basal)	£0.47/day	300 IU	40 IU	£170.23
Intensified insulin	£0.70/day	300 IU	60 IU	£256.96
Diabetes-related complication cost^l^	Fatal	Non-Fatal	Maintenance	Source
Ischaemic heart disease	-	£3,479	£1,149	Clarke, 2003 [21]
Myocardial infarction	£2,244	£6,709	£1,105	Clarke, 2003 [21]
Congestive heart failure	£3,880	£3,880	£1,360	Clarke, 2003 [21]
Stroke	£5,658	£4,103	£776	Clarke, 2003 [21]
Amputation	£13,359	£13,359	£771	Clarke, 2003 [21]
Blindness	-	£1,752	£742	Clarke, 2003 [21]
End-stage renal disease	-	£34,806	£34,806	Baboolal, 2008 [40]
Adverse event, renal monitoring and discontinuation costs		Cost input	Source	
Severe hypoglycaemic event		£390	Hammer, 2009 [41]	
Renal monitoring		£38.67	Assumed to incur one GP visit cost and a 24-hour creatine clearance determination [42, 43]	
Urinary tract infection, genital infection		£36	Assumed to incur one GP visit cost [42]	
Discontinuation		£36	Assumed to incur one GP visit cost [42]	

^a^Value was applied as the HbA1c switching threshold; it was considered to be a representative threshold value in real-world UK clinical practice as it was the average baseline HbA1c value of patients entering the phase 3 clinical trials that were included in the indirect comparison before switching to dual oral therapy

^b^Value sourced from randomised controlled trial by Nauck et al., 2010 [19]

∆ Mean change from baseline

^c^ Effects apply to the first year after treatment initiation

^d^ No estimate available and/or zero value assumed

^e^Probability of discontinuation was applied during the first model cycle

^f^Probabilities of adverse events were applied during every model cycle

^g^Monami, 2008 [44]

^‡‡^NICE HTA report Chapter 4 [45]

^h^Weight change from Montanana, 2008 [46]. Chosen as most recent study reporting weight effect included in the NICE HTA report

^i^The daily costs are based on pack costs and have been rounded. The source of the unit costs are England and Wales Drug Tariff costs, February 2012. These costs are in general consistent with BNF63 drug prices

^j^Sitagliptin is the most frequently prescribed DPP-4i in the UK (80 % of DPP-4i market as of December 2011, data on file)

^k^The cost of insulin was based on a patient baseline weight of 88 kg, which if it remained stable would equate to an annual cost of £170.23 (and £256.96 for intensified insulin). However, in the model, weight changed over time, hence the actual annual cost of insulin (with dosage according to weight) in the economic analysis varies according to the simulated change in weight. Insulin daily cost per kg = £0.0053, insulin intensified daily cost per kg = £0.008

^l^Prices were indexed to 2011 using the Hospital and Community Health Services Pay & Prices index

Abbreviations: HbA1c, Glycated haemoglobin; SBP, systolic blood pressure; TC, total cholesterol; HDL-C, high-density lipoprotein cholesterol; ∆, absolute change from baseline; GP, general practitioner

### Treatment sequence

The first modelled treatment lines were DAPA + MET and DPP-4i + MET for the intervention and comparator groups respectively. Simulated patients received the allocated therapy until their HbA1c level increased towards a pre-specified threshold limit. This switching threshold was set equal to the mean baseline HbA1c value of patients entering the phase 3 clinical trials included in the NMA, (8.05 %). At this point, patients in the model switched therapy; first to insulin + MET and then to intensified insulin (simulated by increasing the dose by 50 %). Patients then remained on this latter treatment for the remainder of the time horizon and the HbA1c levels progressed according to the UKPDS regression analysis. The treatment duration in the model was determined by a combination of the HbA1c baseline value, the HbA1c treatment effect and the predefined HbA1c treatment switch threshold. Patients also had a separate risk of discontinuing therapy due to tolerability issues during the first cycle.

### Treatment effects

For each treatment effect, a one-year reduction in HbA1c and weight was applied using data for the relative efficacy of DAPA + MET and DPP-4i + MET derived from the previously published NMA of dual therapy RCTs; further detailed information on the methods and results of the NMA has been described elsewhere [6]. The authors of the NMA deemed the included studies to be of good quality [6]. The NMA reports the relative effects of each agent in comparison with other agents whereas in this model we used the absolute changes from baseline for each agent (Table 1). Values presented for subsequent treatments (insulin + MET and intensified insulin) were sourced directly from previous studies (Table 1). The probability of discontinuation in the first cycle after treatment initiation and the respective probabilities of hypoglycaemic events, urinary tract infections and genital infections for each therapy are also outlined in Table 1. Of the studies included in the NMA, only two reported the change in SBP as an outcome [19, 20]. Due to the limited evidence available for treatment effect on cholesterol and SBP, no difference in effect between dapagliflozin and DPP-4i was assumed, so these values were set to zero in the model.

### Costs

Costs included in the model were assessed from a UK health service perspective. A systematic literature review covering economic evaluations of relevance to a UK context for drug interventions for T2DM was undertaken. An overview of cost inputs applied in the model is presented in Table 3. UKPDS 65 [21] cost data, indexed to 2011 (the year the analysis was conducted for NICE) using the Hospital and Community Health Services Pay and Price index, was utilised as a key source for model inputs. Acquisition costs were sourced from the NHS Drug Tariff and were regarded as representative of the actual costs paid by the NHS [22].

### Health-related quality of life

A systematic literature review was carried out to identify sources of utilities for those factors that most affect HRQoL in patients with T2DM, namely diabetes-related complications, hypoglycaemia, weight change and other adverse events. The UKPDS 62 study [23] identified from the systematic review was used to inform the majority of values as the utilities were derived from a UK population and this was the same cohort from which the risk equations were derived. Utility data for end-stage renal disease (ESRD), hypoglycaemic events and urinary tract infection were sourced from alternative studies identified in the review [24, 25]. Body weight utilities were sourced from a Canadian study by Lane et al. as this was the only reference identified by the systematic review that made a distinction in terms of utility change for BMI increase and decrease, and the data was elicited specifically from T2DM patients [26] (Table 1). No utility decrement could be identified specifically for genital infections, so this was assumed to be equivalent to that for urinary tract infections.

### Approach to sensitivity analysis

To assess the impact of uncertainty on the model results, both deterministic univariate sensitivity analysis (SA) and probabilistic sensitivity analysis (PSA) were carried out. Parameters selected for variation in the univariate SA were the risk factors known to influence outcomes in the UKPDS equations, as well as others where the uncertainty around the point estimate was high, such as the utilities and the cost of complications. These parameters were varied in the univariate SA around their 95 % confidence/credible intervals. Where data was unavailable, the standard error (SE) was assumed to be a percentage of the mean in line with the magnitude of other SEs for other similar variables. As such, disutilities for T2DM complications were varied by ±10 %, and total non-drug costs were varied by ±25 %. PSA was conducted by simulating 1,000 cohorts of 30,000 patients in which values of key parameters (including those not varied in the deterministic analysis) were drawn randomly and independently from the parameter distributions.

The impact of the HbA1c switching threshold was tested separately, as it helps to determine the treatment duration for each intervention in the model. As the treatment effect on clinical parameters was only applied during this treatment period, it was important to fully test the assumptions made in the calculation of this parameter. The effect on weight was also expected to be an important driver of the cost-effectiveness results, given the impact that DAPA + MET has been reported to have from clinical trials on weight and the significant difference in this outcome between DAPA + MET and DPP-4i + MET reported in the indirect comparison [6, 15, 16]. As such, the utilities associated with this variable were investigated in scenario analysis.

## Results

The treatment group, DAPA + MET, was associated with a mean incremental benefit of 0.032 QALYs (95 % CI: −0.022, 0.140) when compared with the DPP-4i + MET control arm (Table 2). This effect is largely explained by differences in patient weight, which has a significant impact on HRQoL [26]. The mean incremental cost was estimated to be £216 (95 % CI: £-258, £795) and was mainly driven by the higher acquisition cost of dapagliflozin (Table 2). An ICER point estimate of £6,761 per QALY gained was calculated.

**Table 2:** Discounted base case results

Technologies	Total			Incremental			ICER (£)
Add-on to MET	Costs (£)	LYs	QALYs	Costs (£)	LYs	QALYs	Incremental cost per QALY gained
DPP-4i	£13,593	14.80	11.83	-	-	-	-
Dapagliflozin	£13,809	14.80	11.86	+£216	0.01	+0.032	£6,761

Abbreviations: LYs, life years; QALYs, quality-adjusted life years; ICER incremental cost-effectiveness ratio

**Fig. 1:**
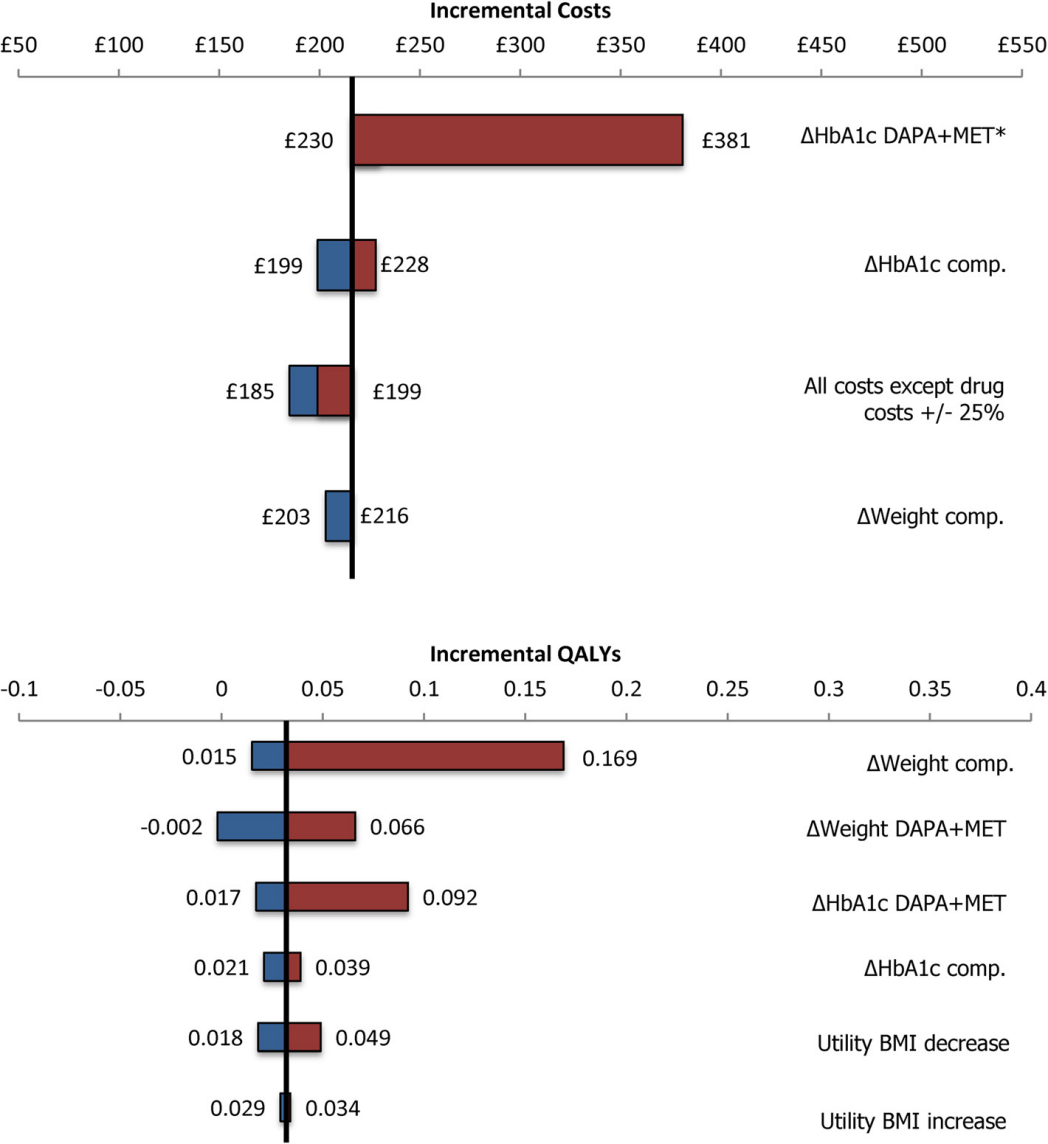
Univariate sensitivity analyses: Tornado graphs of incremental costs (top) and incremental QALYs (bottom). Variations of selected parameters are displayed as a range from the base case value (y-axis). Parameters include HbA1c change from baseline (∆HbA1c), weight change from baseline (∆Weight), BMI utility values and total non-drug costs. *It can be observed from the tornado graph for incremental costs that assuming a larger/smaller effect of dapagliflozin on HbA1c reduction would result in increased incremental costs. This can be explained by the model structure: in case of larger HbA1c reduction, patients would remain on the more expensive treatment option longer, whereas for the smaller HbA1c effect, patients would switch sooner to the next treatment line, leading to increased costs associated with AEs. Abbreviations: Comp., comparator; DAPA + MET, dapagliflozin added on to metformin; QALY, quality-adjusted life year; BMI, body mass index

The results of the univariate SA are presented as tornado graphs (Fig. 1). These highlight the range of both the incremental costs and incremental QALYs for the parameters that most affect these outcomes. The base case value is represented by the central vertical line and the outcome values are plotted for the maximum and minimum values of each selected parameter. As can be seen from Fig. 1, the point estimate for incremental costs was relatively insensitive to the variation applied to the model parameters. Improving the HbA1c lowering effect of dapagliflozin resulted in an incremental cost increase of £165 compared with the base case, due to the increased treatment duration, which eventually led to higher drug acquisition costs for the dapagliflozin strategy. However, due to the incremental gain in QALYs observed (0.06), the overall ICER decreased to £4,140. The point estimate for incremental QALYs was shown to be most sensitive to variation of the treatment effect on body weight of DPP-4i (Fig. 1). When varying the weight effect of DPP-4i between the outer limits of its 95 % CI, the incremental QALYs ranged from 0.015 to 0.169; changing this parameter for dapagliflozin resulted in a QALY range of −0.002 to 0.066.

The assumption around the HbA1c switching threshold was investigated by increasing the value from 8.05 to 8.5 % as studies have shown that many patients will exceed this initial threshold in practice [27, 28]. The resulting ICER decreased from £6,761 to £5,227 per QALY gained. However, by using alternative, lower estimates for BMI utility effect [29], the ICER increased to £12,763 per QALY gained. In both cases, the ICERs still fell below the lower limit of generally accepted ICER values in the UK for diabetes medicines (£20,000 per QALY).

**Fig. 2:**
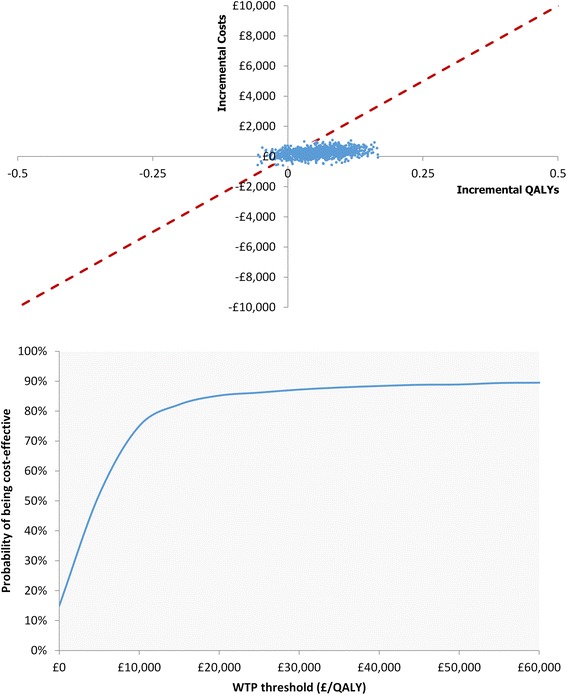
Cost-effectiveness plane of the ICER estimates (top) and cost-effectiveness acceptability curve (bottom) from the PSA for DAPA + MET versus DPP-4i + MET. ICER threshold at £20,000 is represented by the red dashed line in the top panel. Analysis of the PSA result suggested that DAPA + MET had an 85 % probability of being cost-effective at this threshold.

The distribution of the ICER estimates from the PSA shows that in most instances, DAPA + MET is both more effective and more costly than DPP-4i + MET (Fig. 2, top panel). Analysis of the PSA results demonstrated that DAPA + MET had an 85 % probability of being cost-effective compared with the DPP-4i + MET treatment strategy at a willingness-to-pay threshold of £20,000 per QALY gained (Fig. 2, bottom panel).

## Discussion

The economic analysis of DAPA + MET versus DPP-4i + MET, as accepted by NICE in the UK, has shown dapagliflozin to be a cost-effective use of NHS resources and therefore a valuable treatment option for T2DM patients who are inadequately controlled on metformin monotherapy. Results from a previously published NMA showed dapagliflozin added on to metformin resulted in a superior weight reduction outcome compared with DPP-4i [6]. This outcome has been shown to be important to patients in terms of quality of life and was a key driver in the associated gain in QALYs observed with dapagliflozin in this economic evaluation [30]. Ultimately, the results show that the incremental QALYs come at an acceptable incremental cost when employing the commonly accepted payer willingness-to-pay threshold of £20,000 per additional QALY gained employed in the UK by NICE, indicating the cost-effectiveness of dapagliflozin versus DPP-4i as an add-on to metformin for the treatment of T2DM.

The model has previously undergone peer-review [11, 12] and health technology assessments [31] and in terms of structure is very similar to a previously developed and validated cost-effectiveness model [32, 33]. The same model has also been used to compare the cost-effectiveness of DAPA + MET with sulphonylurea + MET; this analysis produced a similar magnitude of costs and QALYs for DAPA + MET as estimated in the current analysis and also concluded that DAPA + MET was a cost-effective use of NHS resources in the UK setting [7]. In addition, the cost-effectiveness of dapagliflozin as an add-on to insulin has been investigated from a Dutch healthcare perspective, where it was also found to have an ICER within acceptable cost-effectiveness limits [8].

Although the model has been validated against UKPDS datasets and extensive scenario and sensitivity analyses have been performed, several assumptions exist which may have an impact on the robustness of the study outcomes. Firstly, the model does not include less severe health states, such as microalbuminuria and foot ulcers; however, the utility and cost impact of such health states would be expected to be minimal and therefore have a negligible impact on the final ICER. Secondly, the model treats the patients as a cohort with mean baseline values and mean treatment estimates, as data availability did not allow for a more sensitive approach. Heterogeneity in the population was tested in the model through the sensitivity analysis, but future modelling could simulate individual patients with different characteristics and link these to treatment effect if clinical trial data allowed. Thirdly, the assumption that the mean HbA1c at baseline is a valid representative of the switching threshold does not take into account the potentially skewed nature of this parameter. Unfortunately, data was not available to allow a normality test; however, the switching threshold has been tested in one-way sensitivity analysis and was found not to change the conclusion over the cost-effectiveness of DAPA + MET over DPP-4i + MET.

In terms of limitations in the clinical inputs, the main one was the lack of an RCT directly comparing DAPA + MET with DPP-4i + MET. In the absence of such a head-to-head trial, an NMA was conducted using Bayesian methodology, the methods and limitations of which have been previously discussed in the publication of Goring et al. [6] The uncertainty around the efficacy of dapagliflozin was investigated through one-way sensitivity analysis and showed that as the efficacy of dapagliflozin on lowering HbA1c levels was increased, the total costs were also increased due to the longer treatment period estimated. The additional QALYs gained, however, mean that the resultant ICER still fell as would be expected. Additionally, there was a lack of available long-term data regarding the effect of dapagliflozin or DPP-4i as an add-on to metformin on the development of diabetes-related micro- and macro-vascular complications; it was assumed instead that valid lifetime predictions of events can be made using the UKPDS 68 risk equations [13]. These risk equations have been widely used by researchers modelling diabetes treatments [10, 11, 33] and although they are not without limitations [34], there are no other sources for risk prediction that have been based on such a large number of T2DM patients. The UKPDS risk equations are also derived from a study of over 5,000 UK patients, making them highly applicable to the perspective of the current analysis. Since this analysis for NICE was performed, more recent UKPDS risk equations than the UKPDS 68 ones have been made available [35], but their use in health economic models has not yet been validated by health technology assessment agencies. Therefore we decided to maintain the use of the UKPDS 68 equations in the model, as these have been reviewed and accepted by NICE during the appraisal of dapagliflozin [36]. We also acknowledge that an alternative source of utility values has been published [37]; however, we think it is important that the model that was reviewed by NICE is published.

Assumptions to extrapolate HbA1c beyond trial outcomes were designed to best represent the progressive nature of T2DM in clinical practice, and the time paths were shown to be in line with those reported in the UKPDS 68 study [13]. Weight change, which was associated with CV risk and a decreased HRQoL whilst on treatment, was extrapolated beyond the 2-year trial data and was shown to be a key risk factor in determining QALYs during the sensitivity analysis. However, as mentioned previously, a conservative approach was adopted when extrapolating these data, and as such, any beneficial effect associated with weight loss was likely underestimated. Recently published long-term data reporting sustained weight effect over 4 years confirms the conservative nature of this assumption [18].

Key uncertainties within the economic evaluation arose around the BMI utilities, as there is some uncertainty over the precise relationship between change in BMI and disutility in T2DM. The values used to estimate the impact of increasing/decreasing BMI on utilities were seen to vary in the literature, although sensitivity analyses showed that this did not impact on the cost-effectiveness of dapagliflozin. The outcomes from the NMA, which included international studies, can be considered to be generalisable to patients in a UK setting as the populations defined were representative of the treatment indication in the UK and the average baseline demographics were similar to the UK T2DM population recruited for the UKPDS studies [13]. Additionally, cost and resource use was derived from UK sources, and utility data were largely sourced from the UKPDS studies. The results may also be of interest to other countries where DPP-4 inhibitors are commonly used in clinical practice as add-ons to metformin, especially as the treatment effect data are sourced from international trials. However, available treatments and advised strategies may differ between countries, and factors such as the risk equations, utilities and costs may be subject to change. As such, extrapolation of these results to countries outside of the UK should be performed with caution and local adaptation of the economic model would be advised.

## Conclusion

Dapagliflozin represents the first-in-class selective SGLT2 inhibitor licensed in Europe, and has shown in clinical trials to have an effect on HbA1c comparable with existing treatments and a superior outcome in terms of weight reduction [38]. This analysis confirmed that DAPA + MET, in comparison to DPP-4i + MET, is cost-effective within acceptable UK thresholds for the treatment of patients with T2DM.

## Availability of supporting data

Supporting data used for the development of the model is available on request.

## Abbreviations

ADA: American Diabetes Association
BMI: Body mass index
CV: Cardiovascular
DAPA: Dapagliflozin
DPP-4i: Dipeptidyl peptidase-4 inhibitor
ESRD: End-stage renal disease
EASD: European Association for the Study of Diabetes
HbA1c: Glycated haemoglobin
HRQoL: Health-related quality of life
HDL: High-density lipoprotein
ICER: Incremental cost-effectiveness ratio
MET: Metformin
NHS: National Health Service
NICE: National Institute for Health and Care Excellence
PSA: Probabilistic sensitivity analysis
QALYs: Quality-adjusted life years
RCTs: Randomised controlled trials
SA: Sensitivity analysis
SGLT2: Sodium-glucose co-transporter 2
SE: Standard error
SBP: Systolic blood pressure
T2DM: Type 2 diabetes mellitus
UKPDS: UK Prospective Diabetes Study
